# Evaluation of Pulsed Light to Inactivate *Brettanomyces bruxellensis* in White Wine and Assessment of Its Effects on Color and Aromatic Profile

**DOI:** 10.3390/foods9121903

**Published:** 2020-12-19

**Authors:** Antonio J. Pérez-López, María I. Rodríguez-López, Francisco Burló, Ángel A. Carbonell-Barrachina, José A. Gabaldón, Vicente M. Gómez-López

**Affiliations:** 1 Departamento de Ciencia y Tecnología de Alimentos, Universidad Católica de Murcia (UCAM), Campus de los Jerónimos 135, 30107 Guadalupe, Spain; ajperez@ucam.edu (A.J.P.-L.); mirodriguez@ucam.edu (M.I.R.-L.); jagabaldon@ucam.edu (J.A.G.); 2 Departamento de Tecnología Agroalimentaria, Escuela Politécnica Superior de Orihuela (EPSO), Universidad Miguel Hernández de Elche (UMH), 03312 Orihuela, Spain; francisco.burlo@umh.es (F.B.); angel.carbonell@umh.es (Á.A.C.-B.); 3 Cátedra Alimentos para la Salud, Universidad Católica de Murcia (UCAM), Campus de los Jerónimos 135, 30107 Guadalupe, Spain

**Keywords:** pulsed light, wine, *Brettanomyces bruxellensis*, color, volatile compounds

## Abstract

*Brettanomyces bruxellensis* is a wine spoilage yeast that could be inactivated by pulsed light (PL); however, this technology may induce changes in the quality of this alcoholic drink. The present research aimed to determine the potential of PL to inactivate *B. bruxellensis* inoculated in white wine and to assess the effect of this technology on the color and aromatic profile of the wine. For this, a cocktail of *B. bruxellensis* strains was inoculated into the wine and its inactivation by PL was determined and fitted to a microbial inactivation model. Along with this, the effect of PL on instrument-measured color, and the volatile compounds of the wine were evaluated by GC/MS and descriptive sensory analysis, respectively. *B. bruxellensis* was inactivated according to the Geeraerd model including the tail effect, with a maximum inactivation of 2.10 log reduction at 10.7 J/cm^2^; this fluence was selected for further studies. PL affected wine color but the total color difference was below the just noticeable difference at 10.7 J/cm^2^. The concentration of 13 out of 15 volatile compounds decreased due to the PL, which was noticeable by the panel. It is not clear if these compounds were photolyzed or volatilized in the open reactor during treatment. In conclusion, PL is able to inactivate *B. bruxellensis* in white wine but the treatment impairs the volatile profile. The use of a closed reactor under turbulent flow is recommended for disaggregating yeast clumps that may cause the tailing of the inactivation curve, and to avoid the possible escape of volatile compounds during treatment.

## 1. Introduction

Usually, but not exclusively, white wine is made from white grape musts. The main process by which must is transformed into wine is alcoholic fermentation, which consists of the transformation of the sugars (glucose and fructose) contained in grapes into ethanol and carbon dioxide [1]. For wine production, white wine grapes are destemmed, crushed and pressed, so that they do not macerate, thus avoiding the extraction of bitter and easily-oxidizable polyphenols. White wines take on all the nuances of the yellow color spectrum from the very pale with greenish hues, to hues that are slightly copper and orange [2].

In general, and as with red wines, most white wines are usually classified according to the grape used (variety, *Botrytis* grapes, late harvest, etc.), sugar levels (dry, semi-dry, or sweet), elaboration procedure (fermentation in barrels, aging “on lees”, aging in barrels, sparkling wines, etc.), or by its aging techniques (oxidative in barrels, inert in tanks, etc.) [3]. The Macabeo white grape variety (*Vitis vinífera* L. var. Macabeo) is commonly used for wine production and is found on both sides of the Pyrenees: in the north and east of Spain, and in the south of France. This variety grows well in hot, dry regions. It also sprouts later, which makes it less likely to be damaged by frost. It is a versatile grape, and is used in sparkling, sweet, and dry wines. In terms of flavor, the wine can be fresh, floral, and aromatic when harvested at the beginning of maturation, or it can be dense with a honey flavor when picked a little later at maturation and aged in oak barrels. It is not a very aromatic grape, and its relative neutrality makes it a good candidate for blending. It can show grapefruit flavors and high acidity when harvested early. However, in late harvests, the oak flavor predominates over the other flavors in these wines. It produces a wine with a delicate aroma and a pale straw-yellow color with green tones. They are not usually very alcoholic wines, with an alcohol content between 9 and 11% [4].

The Alicante Protected Designation of Origin has the privilege of having a wine-producing area with very particular characteristics, as it benefits from an average of 2800 h of clear sun that directly affects the crop’s evapotranspiration. In the Marina Alta area, a production area for the Macabeo variety, the vineyards are located near the sea and benefit from the daily Mediterranean breeze. Due to the rugged orography of this area, the vineyards are organized in terraces. The average altitude of the vineyards is 600 m. The soils are mainly limestone, healthy and without the presence of organic matter. The climate is Mediterranean tending to continental inland, with a level of rainfall of about 500 mm [5].

The spoilage of wine by *Brettanomyces bruxellensis* is a major problem in the wine industry. As recently reviewed by Cibrario et al. [6], this yeast produces volatile phenols: 4-vinylphenol, 4-vinylguaiacol, 4-ethylphenol and 4-ethylguaiacol, which results in unpleasant aromatic notes (such as animal, leather, horse, stable, or pharmaceutical), and are often referred to as the “brett character”. The aromatic note is the term used in oenology to describe an aromatic impression that reminds us of an odor characteristic of a determined source. Sulfites have been traditionally used to avoid wine spoilage, however, aside from the recent trend to eliminate their use, the existence of *Brettanomyces* strains resistant to sulfur oxide (SO_2_) has been demonstrated [7].

Pulsed light (PL) is an emerging non-thermal technology for microbial inactivation that consists of the use of high-intensity short-pulses of polychromatic light with wavelengths spanning from 200 to 800 nm, where the UV-C portion accounts for most of its antimicrobial efficacy [8]. It has a broad spectrum of antimicrobial action, from viruses to parasites. It has the advantages of being a non-thermal technology that can inactivate microorganisms very quickly, usually in a few seconds, and it leaves no residues. On the other hand, given its poor penetrability, its efficacy is limited to food, food-contact surfaces, and transparent liquids [8]. *B. bruxellensis* can be inactivated by pulsed light (PL), as shown in previous works where the efficacy of this technology was demonstrated when used to inactivate yeasts such as *Rhodotorula mucilaginosa* [9], different *Candida* species [10], and *Saccharomyces cerevisiae* [11]. PL inactivates yeasts by causing DNA and cell membrane damage [12].

The problems associated to *Brettanomyces* growth are more common in red wines, but white wines are not exempt from them [13], and are more suitable for a first *Brettanomyces* inactivation attempt through the use of a new photonic technology due to their higher transparency in comparison with red or rose wines. Indeed, UV-C light technology, a method closely related to PL technology, has only been tested against *B. bruxellensis* in white wines [14,15].

Even though PL may have a beneficial effect on the stability of wines by decreasing the contamination level of spoilage yeasts, this benefit may be limited by the potential harmful effects on wine quality. To the best of our knowledge, the application of PL to wines has not been reported up until now.

The goal of this research was to test the potential of pulsed light technology for inactivating *Brettanomyces bruxellensis* in white wines, and to assess the possible detrimental effect that this treatment could have on the quality of the wine, more specifically, on the wine’s color and aromatic profile.

## 2. Materials and Methods

### 2.1. Wine Samples

The white wine samples were obtained from a 2018 harvest. Only grapes of the Macabeo variety were used for their production, and all these samples were provided by the BOCOPA (Preter, Alicante, Spain) wine company, which belongs to the Alicante Protected Designation of Origin. The samples were collected in triplicate directly from the barrels after wine stabilization. Wine, simply fermented and not aged, was physico-chemically characterized according to the official methods from the International Organization of Vine and Wine (OIV) [16]. The wine had the following characteristics:

- total alcohol content: 12% (ABV)
- total acidity: 4.87 g tartaric acid/L
- volatile acidity: 0.24 g acetic acid/L
- relative density at 20°: 0.9991
- total dry extract: 17.3 g/L
- reducing sugars:1.6 g/L
- total SO_2_: 106 mg/L
- pH: 3.3.

### 2.2. Yeast Strains

Two *Brettanomyces bruxellensis* strains were used in this study: CECT 1009 and CECT 1010, which were purchased from the Spanish Type Culture Collection (CECT) (Paterna, Valencia, Spain). The strains were activated in yeast-peptone-dextrose (YPD) broth (Sigma, Saint Louis, MO, USA) at 26 °C for 10 days according to CECT instructions. *Brettanomyces* cultures were stored in tryptic-soy agar (TSA) (Sigma, Saint Louis, USA) at 4 °C for up to three months.

In order to carry out the tests, the strains were processed separately until mixed in the wine sample. They were grown separately in 10 mL of YPD Broth and incubated for 10 days at 26 °C. Subsequently, 1 mL of these cultures were added to a 100 mL YPD Broth flask and incubated for 4 days at 26 °C with shaking at 120 rpm, and then centrifuged (2900× *g*, 10 min) to collect the yeast cells. The pellets were washed twice with the wine, obtaining a final concentration of 10^7^ CFU/mL according to a plate count, and then resuspended in 45 mL of wine. Finally, 1 mL of each strain suspension was added to 18 mL of wine and treated with PL.

### 2.3. Pulsed Light Treatment

The pulsed light treatment was carried out using a XeMaticA-Basic-1L unit (Steribeam, Germany) operated at 2.5 kV. The emission spectrum of this system, utilized under similar operating conditions, has been reported before [17]. The reactor was a rectangular parallelepiped that was 20 cm wide, 14 cm high, and 10 cm deep. It had a 19 cm-long xenon lamp placed at the top of the chamber. The sample was placed 7.1 cm below the center of the lamp. Since the lamp was not surrounded by the wine, the system can be classified as an open photoreactor [18]. Twenty milliliters of inoculated wine samples were placed in a 90 mm diameter Petri dish and illuminated with pulses of 2.14 J/cm^2^ measured at the sample surface. Pulse fluence was determined via a built-in photodiode signal analyzer using an oscilloscope and manufacturer performance charts as previously described [19]. Fluence is the amount of light energy impinging a sample per unit of sample surface. A formal definition of this term and its application to pulsed light tests can be found in the glossary of terms of the International Union of Pure and Applied Chemistry [20], and Gómez-López and Bolton [21]. A maximum of 20 pulses were utilized (42.8 J/cm^2^), and the sample was shaken between pulses. After a predetermined number of pulses, one mL of wine sample was taken, seeded in Brettanomyces Agar (Scharlau, Barcelona, Spain), and incubated for 6–8 days at 26 °C. All the samples were made in duplicate and the experiments were repeated three times.

### 2.4. Brettanomyces bruxellensis Inactivation Kinetics

To describe the inactivation kinetics of *B. bruxellensis*, the data obtained after the PL treatments was used to fit different models with fluence utilized as the independent variable. Models were fitted with GInaFIT software version 1.6 (Katholieke Universiteit Leuven, Belgium), a free Microsoft^®^ Excel program [22], and the fit of the models to the data was evaluated using the root mean square error (RMSE) as recommend by Geeraerd et al. [22]. The Geeraerd model including the tail effect was selected for further analysis. This model describes the shape of a microbial inactivation curve that has an initial log-linear phase followed by a tail, with tailing being defined as a phase where the microbial population ceases to decrease. The model reads as:

(1) $$\log N_{F}=\log(10^{\log N_{0}}-10^{\log N_{\mathrm{res}}})\;e^{-\;k_{\max}\;F}+10^{\log N_{\mathrm{res}}}$$

where N_F_ is the population (log CFU/mL) at fluence F (J/cm^2^), N_0_ is the initial population (log CFU/mL), N_res_ is the residual population (log CFU/mL) and k_max_ is the maximum specific inactivation fluence (cm^2^/J).

### 2.5. Color Determination and Absorbance Spectrum

The color of the white wine was determined using a ColorFlex 45/0 device (Hunterlab, Virginia, USA). Measurements were carried out at room temperature according to the CIE (Committee International d’Elairage) Lab color notation system. The instrument was calibrated with standard white (L* = 93.17, a* = −0.96, b* = 1.53) and black (L* = 0.29, a* = 0.47, b* = 0.05) tiles, and wine samples were measured in a glass dish (25 mm diameter). Hue angle (h^o^) was calculated from tan^−1^ (b*/a*), and Chroma was calculated as (a*^2^ + b*^2^)^½^. The total colorimetric difference between the color of treated and untreated wine samples is given by the CIELAB color difference [23]:

ΔE* = ((L_F_* − L_0_*)^2^ + (a_F_* − a_0_*)^2^ + (b_F_* − b_0_*)^2^)^½^ (2)

where L_0_*, a_0_* and b_0_* are the color parameters of the untreated samples, and L_F_*, a_F_* and b_F_* are the color parameters of samples treated at fluence F.

The UV spectrum of the wine was determined in a 1:20 dilution of wine in demineralized water measured in a quartz cuvette with a 1 cm optical path length using a spectrophotometer (Shimadzu UV-1603, Japan). Dilution was required because the absorbance spectrum of the undiluted sample could not be measured due to excessive light absorption.

### 2.6. Aromatic Profile of Wine

The volatile composition of wines was obtained through headspace solid phase microextraction (HS-SPME) following the methodology previously reported by Issa-Issa et al. [16], and Zapata et al. [24]. Fifteen milliliters of wine were placed into 50 mL vials with polypropylene caps and PTFE/silicone septa. A magnetic stirring bar and 1.5 g of NaCl were added, and then the vial was placed in a water bath at 45 °C for 50 min. During this time, a 50/30 µm DVB/CAR/PDMS fiber (Supelco, Bellefonte, PA, USA) was exposed to the sample headspace.

The identification and semi-quantification of the volatile compounds were performed on a Shimadzu GC-17A gas chromatographer (GC-MS), (Shimadzu Corporation, Kyoto, Japan), coupled with a Shimadzu GC-MS QP-5050A mass spectrometer detector. The column used was a Restek Rxi-1301 Sil MS (Restek Corporation, Palo Alto, CA, USA) measuring 30 m in length, 0.25 mm internal diameter, and 0.25 µm film thickness. Analyses were carried out using helium as a carrier gas at a flow rate of 6 mL/min according to the following programmed temperature: initial temperature 80 °C; rate of 3.0 °C/min to 210 °C, and hold for 1 min; rate of 25 °C/min from 210 to 300 °C and hold for 3 min. The analysis was carried out from 39 to 400 m/z, with an electronic impact (EI) of 70 eV, in 1 scan/s mode. Desorption of the volatile compounds from the fiber coating was carried out in the injection port (230 °C) for 3 min. The temperature of the detector was 300 °C. Benzyl acetate (1000 ppm) was used as internal standard.

Most of the compounds were simultaneously identified by using three different analytical methods: (i) retention indices, (ii) GC-MS retention times (authentic chemicals), and (iii) mass spectra (Wiley spectral library collection). The retention indexes were calculated using standards of aliphatic hydrocarbons in the range from C–5 to C–23.

The volatile composition analysis was run in triplicate and results were expressed as concentration (mg/L) of each volatile compound.

### 2.7. Descriptive Sensory Analysis with Trained Panel

The sensory characteristics of untreated wine and wine treated with PL at the selected fluence (10.7 J/cm^2^) were compared. Twelve panelists (6 males and 6 females), aged 25–62 years, evaluated the wine samples at the facilities of the Universidad Miguel Hernández of Elche (UMH). Each of the panelists had more than 500 h of testing experience with wine samples. The questionnaire and lexicon used were described by Issa-Issa et al. [16].

Thirty-five milliliters of wine were served in a black cup for the analysis of the scent (nasal perception of volatile compounds), flavor (a combination of scents, aroma (retronasal perception of volatile compounds), basic tastes, and chemical feeling factors), global attributes, and for the wine’s appearance; in this case, 25 mL of wine were served in a transparent cup. Samples were evaluated at 10 °C.

The temperature of the testing room was set at 21 °C; the illumination was a combination of natural and non-natural (fluorescent) light. Samples were randomly served coded with 3-digit numbers together with the appropriate questionnaire, one at a time, and with a seven minutes wait between samples. Between samples and for palate cleansing, unsalted crackers and water were provided to panelists. The attributes under evaluation were: aroma (alcohol, fruity, floral, citrus, white flowers, vegetable, spicy, animal, and toasted), flavor (alcohol, fruity, floral, vegetable, spicy, animal, toasted, sweet, sour, bitter, and astringent), global attributes (imbalances and aftertaste), appearance (limpidity and color) and defects (rotten apple, vinegar, glue, soap, sulfur, rotten egg, onion, cauliflower, horse, earthy, and cork).

Panelists used a scale ranging from 0 to 10 points for the evaluation, where 10 was extremely high intensity and 0 was extremely low intensity or not noticeable.

### 2.8. Statistical Analysis

Mean values were analyzed with a one-way ANOVA and according to Tukey’s test (*p* < 0.05) using IBM SPSS Statistics 24 (Armonk, NY, USA). All experiments were carried out in triplicate.

## 3. Results and Discussion

### 3.1. Inactivation of Brettanomyces bruxellensis Inoculated into White Wine

**Figure 1:**
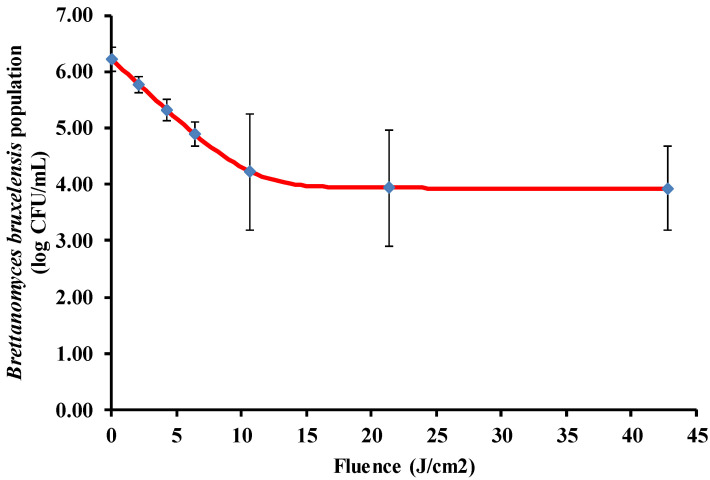
Inactivation of *Brettanomyces bruxellensis* in white wine by pulsed light. The line shows the fitting of the Geeraerd model including the tail effect.

Pulsed light was able to inactivate *B. bruxellensis* inoculated into white wine (Figure 1). The inactivation occurred very quickly at the beginning of the treatment, with a 2.10 log reduction observed after only five pulses (10.7 J/cm^2^), after which tailing was observed. There are PL devices currently on the market that can work at pulse repetition rates as fast as three pulses per second [25], which means that the maximum inactivation rate could be reached in two seconds at industrial scales. The level of inactivation may be sufficient to stabilize wines, given that *B. bruxellensis* is generally present in low amounts in wines, and high population levels are required for volatile phenol production. For example, as a general trend, counts ≤1.92 log cells/mL were detected in 22 Albanian bottled wines [26], and the level of contamination observed in 13 different bottled wines from the Bordeaux area was <2 log cells/mL [27]. Furthermore, it is known that for volatile phenol production to be triggered, a high concentration of *B. bruxellensis*, 10^5^–10^6^ cells/mL is required [28,29].

The Geeraerd model including the tail effect of microbial inactivation, fit well with our data (RMSE = 0.77), and the corresponding constants were: k_max_ = 0.50 ± 0.18 cm^2^/J, log N_0_ = 6.22 ± 0.35 log CFU/mL and log N_res_ = 3.93 ± 0.34 log CFU/mL. Several explanations have been provided to understand the tailing phenomenon that is frequently observed in microbial photoinactivation. The most recent theory explains tailing as a consequence of the photoprotective effect of Mie scattering due to microbial clusters whose frequency has a Gaussian distribution; therefore, a suitable method for cluster disaggregation may result in improved inactivation if this is sought [30]. To the best of our knowledge, there are no studies about the effect of PL on wines, although studies have been conducted on the effect of a similar preservation method, UV-C light. This technology has been able to reduce *B. bruxellensis* counts by 4.8 logs in Chardonnay wine [14]. This higher efficacy may be a result of the type of reactor utilized. While the current work was carried out in a batch system, the work by Fredericks et al. [14] was performed in a continuous turbulent reactor that allows for a better exposure of the microorganisms to light; however, the effect of this system on the quality of the wine was not assessed. A similar efficacy of UV-C on *B. bruxellensis* has been reported in Sauvignon white wine using a coiled reactor [15].

**Figure 2:**
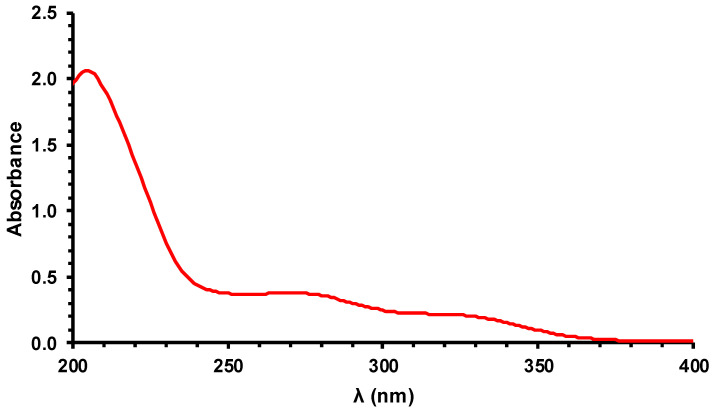
UV absorption spectrum of white wine (diluted 1:20).

The microbicide effect of PL depends on the light reaching the microorganisms; therefore, knowing the spectral absorption characteristics of the sample allows an understanding of how much it limits the effects of this technology. The absorption spectrum of the wine (Figure 2) showed negligible absorbance in the visible range (data not shown). The wine absorbed light in the UV range with a maximum at 204 nm, likely due to absorption by the B-band of phenols present in the wine [31]. There was light absorption at 254 nm, which is generally considered the most germicidal wavelength [21]. This interferes with the antimicrobial efficacy of the treatment, however, the absorbance at this wavelength was not very strong, and it was less than 20% than the absorbance at the peak of the spectrum.

### 3.2. Effect of Pulsed Light Treatment on Wine Color

Aside from the antimicrobial efficacy of a given preservation technology, its effect on product quality should be evaluated. Tristimulus colorimetry was used to evaluate the impact of the PL treatment on the color of white wine. It can be observed (Table 1) that PL affects all color parameters when its application is excessive, as the treatment with a fluence of 42.8 J/cm^2^ promotes statistically significant changes (*p* < 0.05) in all color parameters. However, at the fluence selected as the maximum, according to the microbiological test (10.7 J/cm^2^), only the parameter a* was significantly different (*p* < 0.05). Loss of green color as a consequence of PL treatment has been observed in Green Rosa lettuce [32] and unripe persimmons [33]. The total color difference of the samples increased linearly with fluence with a high correlation (R^2^ = 0.99), and following a relationship ΔE* = 0.062 F, where F is the fluence (J/cm^2^). The ΔE* became higher than the just a noticeable difference (2.3) [34] at fluences > 21 J/cm^2^, which indicates that the color change should not be noticeable to an unexperienced observer at the fluence selected for further analysis (10.7 J/cm^2^).

### 3.3. Effect of Pulsed Light Treatment on Volatile Compounds of Wine

The effect of the PL treatment on the concentration of 15 volatile compounds characteristic of the wine is shown in Table 2. The volatile profile of this wine is mainly composed of esters (isoamyl acetate, ethyl hexanoate, phenylethyl acetate, ethyl octanoate, ethyl 9-decenoate, ethyl decanoate, isoamyl octanoate and ethyl dodecanoate), and terpenes (limonene, linalool, α-terpineol and geraniol). These compounds provide the characteristic smell of white wines such as fruity, citrus, floral, grape, apple, pear, and banana aromas. Ethyl octanoate and ethyl decanoate were the main volatile compounds found in the wine samples. In the case of untreated wine, these compounds represent more than 70% of the total volatile concentration. These compounds are sensorily related to grape and floral aromas [35]. Next, the compounds found in the highest concentration were ethyl hexanoate and octanoic acid. These four compounds are the most common in white wines [36].

The concentration of 13 out of 15 volatile compounds was significantly (*p* < 0.05) decreased by the PL treatment, with the contribution of ethyl octanoate and ethyl decanoate to the total volatile concentration falling to ~50%. It is not clear whether this effect was caused by the photolysis of volatile compounds or due to their volatilization in the open reactor during treatment. There are no closed PL reactors currently available on the market that allow assessment of the latter possibility.

Although there are no studies about the effect of PL on the volatile profile of wines, there are some studies that report its effect on the volatile composition of other foods. In general, PL affects the volatile profile of different products, but with differences on its impact on the quality of the product. Some foods undergo immediate changes, which disappear during storage. This is the case for Gouda and Manchego cheeses, which experience a noticeable increase in sulfur compounds after treatment with PL (≥4.2 J/cm^2^) [37], and it is also the case for Serrano and Iberian hams, in which sulfur and metallic notes appear with a PL treatment of 8.4 J/cm^2^ [38]. PL also caused changes in the volatile profile of fermented mulberry juice, although a sensory analysis revealed that the PL-treated juices were more preferred than the pasteurized juice [39]. It has also been shown that it affects the volatile profile of shiitake mushrooms, specifically the content of C8 compounds contributing to mushroom sensory properties [40]. On the other hand, no effect of PL on selected volatile compounds was observed in non-fat dried milk [41].

**Figure 3:**
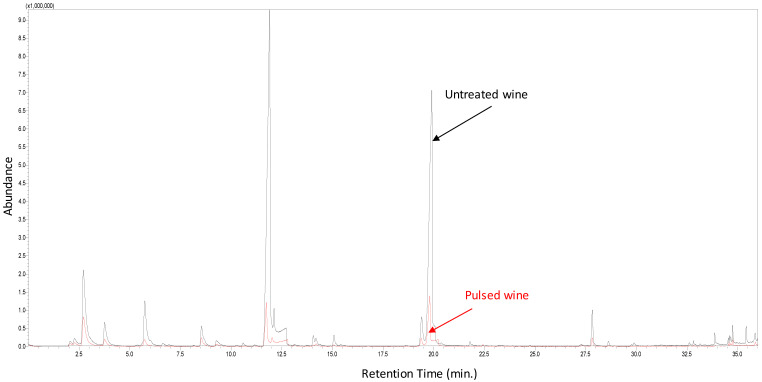
GC-MS chromatograms of untreated and pulsed light treated white wine.

**Figure 4:**
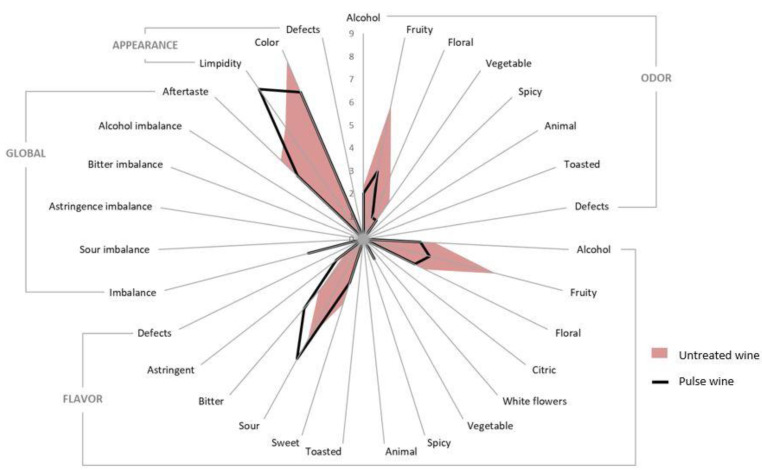
Sensory descriptive analysis of wine samples subjected to pulsed light (10.7 J/cm^2^).

The sensory analysis carried out by a trained panel confirmed the modification of the aromatic profile of the wine observed in the chromatographic results (Figure 3). The alteration of the wine as a consequence of PL treatment showed a decrease in the olfactory levels of fruity and floral aromas (Table 3, Figure 4). The flavor experienced a reduction in fruity, floral and citric notes together with a global imbalance. On the other hand, the limpidity of the wine was improved by the PL treatment. In comparison, no effect on sensorial properties was detected after treating Gros Manseng sweet white wine with UV-C light [15]. The difference could be related to some extent to the use of a coiled reactor, where wine is pumped through a transparent pipe that is coiled around a lamp. This closed reactor avoids the volatilization of compounds, therefore a PL reactor with the same design may yield better preservation of the aroma profile of wines.

## 4. Conclusions

Pulsed light (PL) technology is able to inactivate *Brettanomyces bruxellensis* inoculated into white wine. The inactivation was log-linear with a k_max_ = 0.50 ± 0.18 cm^2^/J and a maximal inactivation of 2.1 log CFU/mL at 10.7 J/cm^2^, followed by tailing, and was fitted by the Geeraerd model including the tail effect. While the treatment does not meaningfully affect the instrumentally-measured total color difference, it perceptibly affected the aromatic profile of the wine. It is uncertain if this effect is due to photochemical reactions or a consequence of volatilization during treatment in an open batch reactor. It is therefore recommended that new tests be carried out in flow-through systems that should become available on the market. PL may provide the wine industry with a technology that would be useful for controlling the risk associated with *Brettanomyces* contamination. However, further studies are required in order to understand if a PL closed reactor can avoid the changes in the aromatic profile found in the wines treated with this technology.

## Figures and Tables

**Table 1:** Effect of pulsed light on the color of white wine.

Fluence (J/cm^2^)	L*	a*	b*	h°	C*
0.0	79.97 ± 0.29 ^b^	−1.70 ± 0.05 ^d^	10.08 ± 0.22 ^c^	−80.36 ± 0.44 ^c^	10.18 ± 0.21 ^c^
2.1	79.97 ± 0.26 ^b^	−1.66 ± 0.04 ^cd^	10.08 ± 0.37 ^c^	−80.63 ± 0.51 ^c^	10.21 ± 0.36 ^bc^
4.3	79.76 ± 0.36 ^b^	−1.62 ± 0.03 ^cd^	10.12 ± 0.34 ^bc^	−80.92 ± 0.43 ^c^	10.25 ± 0.43 ^bc^
6.4	79.71 ± 0.31 ^b^	−1.58 ± 0.04 ^c^	10.12 ± 0.27 ^bc^	−80.68 ± 0.40 ^c^	10.24 ± 0.27 ^bc^
10.7	79.73 ± 0.34 ^b^	−1.57 ± 0.05 ^bc^	10.42 ± 0.21 ^bc^	−80.96 ± 0.44 ^bc^	10.54 ± 0.20 ^bc^
21.4	79.25 ± 0.27 ^ab^	−1.47 ± 0.03 ^b^	10.87 ± 0.31 ^b^	−82.05 ± 0.24 ^b^	10.97 ± 0.30 ^b^
42.8	78.51 ± 0.13 ^a^	−1.32 ± 0.02 ^a^	12.33 ± 0.18 ^a^	−83.90 ± 0.16 ^a^	12.40 ± 0.18 ^a^

L*, lightness; a*, red-green; b*, yellow-blue; h°, hue angle; C*, chromaticity. Within a column, values with different superscript letters are statistically significant (*p* < 0.05) according to Tukey’s test.

**Table 2:** Volatile compounds of wines subjected to pulsed light (10.7 J/cm^2^).

#	Compound	RT (min)	ANOVA ^†^	Untreated Wine	Pulsed Wine	Sensory Descriptor
				Concentration (mg/L)		
1	Isoamyl acetate	3.751	***	0.61 a ^‡^	0.11 b	Banana, pear
2	Ethyl hexanoate	5.736	***	1.16 a	0.09 b	Ethereal, pineapple
3	Limonene	6.636	NS	0.07	0.00	Sweet, citric
4	Linalool	8.542	***	0.47 a	0.11 b	Sweet, citric, floral
5	Phenylethyl alcohol	9.290	**	0.19 a	0.04 b	Honey, rose
6	Ethyl octanoate	11.903	***	8.05 a	0.45 b	Floral, pear, pineapple
7	α-Terpineol	12.119	***	0.56 a	0.10 b	Floral, lilac
8	Octanoic acid	12.714	***	1.17 a	0.28 b	Oily
9	Phenylethyl acetate	14.191	**	0.13 a	0.02 b	Apple, grape, melon, citrus, sweet
10	Geraniol	15.075	**	0.16 a	0.01 b	Floral, fruity, rose, apple
11	Ethyl 9-decenoate	19.400	***	0.56 a	0.08 b	Fruity
12	Ethyl decanoate	19.905	***	6.17 a	0.57 b	Grape, oily, pear
13	Decanoic acid	19.999	**	0.39 a	0.15 b	Waxy, fruity
14	Isoamyl octanoate	21.788	NS	0.05	0.01	Apple, coconut, green, fruity
15	Ethyl dodecanoate	27.828	**	0.45 a	0.06 b	Green, fruity, floral
		TOTAL	***	20.19 a	2.09 b	

^†^ NS: not significant at *p* < 0.05; ** and ***: significant at *p* < 0.01 and 0.001, respectively. ^‡^ Values (mean of 3 replications) followed by the same letter, within the same volatile compound, were not significantly different (*p* < 0.05), Tukey’s least significant difference test [35].

**Table 3:** Sensory descriptive analysis of wine samples subjected to pulsed light (10.7 J/cm^2^).

Attribute	ANOVA ^†^	Untreated Wine	Pulsed Wine
*Odor*			
Alcohol	NS	2.3	2.0
Fruity	***	6.0 ^a ‡^	3.0 ^b^
Floral	***	3.0 ^a^	1.0 ^b^
Vegetable	**	2.0 ^a^	1.0 ^b^
Spicy	NS	0.0	0.0
Animal	NS	0.0	0.0
Toasted	NS	0.0	0.0
Defects	NS	0.0	0.0
*Flavor*			
Alcohol	**	3.1 ^a^	2.5 ^b^
Fruity	***	6.0 ^a^	3.0 ^b^
Floral	**	3.0 ^a^	1.0 ^b^
Citrus	***	2.0 ^a^	0.0 ^b^
White flowers	***	2.0 ^a^	0.0 ^b^
Vegetable	**	2.0 ^a^	1.0 ^b^
Spicy	NS	0.0	0.0
Animal	NS	0.0	0.0
Toasted	NS	0.0	0.0
Sweet	**	3.0 ^a^	2.0 ^b^
Sour	*	5.0 ^b^	6.0 ^a^
Bitter	**	3.0 ^b^	4.0 ^a^
Astringent	NS	1.0	1.5
Defects	NS	0.0	0.0
*Global*			
Imbalance	***	0.0 ^b^	2.5 ^a^
Sour imbalance	***	0.0 ^b^	2.0 ^a^
Astringency imbalance	NS	0.0	0.0
Bitter imbalance	***	0.0 ^b^	2.0 ^a^
Alcohol imbalance	NS	0.0	0.0
Aftertaste	**	5.0 ^a^	4.0 ^b^
*Appearance*			
Limpidity	***	6.0 ^b^	8.0 ^a^
Color	***	8.5 ^a^	7.0 ^b^
Defects	NS	0.0	0.0

^†^ NS: not significant at *p* < 0.05; ** and ***: significant at *p* < 0.01 and 0.001, respectively. ^‡^ Values followed by the same letter, within the same attribute, were not significantly different (*p* < 0.05), Tukey’s least significant difference.
